# Effect of biofilm formation in a hostile oxidative stress environment on the survival of *Campylobacter jejuni* recovered from poultry in Iraqi markets

**DOI:** 10.14202/vetworld.2024.136-142

**Published:** 2024-01-18

**Authors:** Manal H. G. Kanaan

**Affiliations:** Department of Nursing, Technical Institute of Suwaria, Middle Technical University, Baghdad, Iraq

**Keywords:** biofilm, *Campylobacter jejuni*, hostile environment, oxidative stress, poultry meat

## Abstract

**Background and Aim:**

*Campylobacter jejuni* is a major contributor to bacterial enteritis, a common health problem. The resistance of this microaerophilic bacterium to oxidative stress allows it to thrive under aerobic conditions. This study aimed to investigate whether the capacity of *C. jejuni* to form biofilms in the presence of oxidative stress contributes to the pathogen’s ability to thrive in agricultural settings as well as in chicken slaughter lines.

**Materials and Methods:**

Twenty identified strains originating from chicken samples (eight from caeca contents and 12 from frozen chicken carcasses) were previously isolated and identified according to standard bacteriological protocols, followed by confirmation at the species level using multiplex polymerase chain reaction assay. Crystal violet staining was used to evaluate biofilm formation by these bacteria. Two exposure periods to gaseous ozone (1 and 2 min) were used to assess resistance to oxidative damage.

**Results:**

Most of the strong biofilm-forming *Campylobacter* strains came from imported frozen chicken meat (25%), whereas only 10% came from caeca content. After exposure to gaseous ozone at 600 mg/h for 2 min, strong biofilm-producing strains exhibited a higher survival rate with a limited reduction of up to 3 logs, whereas negative biofilm-producing strains exhibited a limited survival rate with a reduction of 6 logs.

**Conclusion:**

Based on our findings, we hypothesized that the presence of *C. jejuni* strains capable of forming biofilms in poultry farms and/or chicken production facilities triggers a public health alarm as this bacterium seems to be able to adapt more easily to live and thrive in hostile environmental conditions.

## Introduction

The zoonotic bacterium *Campylobacter jejuni* causes an estimated 166 million cases of diarrhea and 37,600 deaths annually, making it the most common cause of gastroenteritis in humans [1]. The capacity of *C. jejuni* to live and thrive in all phases of the chicken meat production environment is crucial because the poultry reservoir is often the principal source of infection [2, 3]. However, *C. jejuni* may be collected from any number of environmental sources [2]. Planktonic *C. jejuni* is particularly sensitive to numerous stressors [4, 5], notably oxygen pressure in the environment, which further complicates its ability to induce foodborne diarrheal disease [6, 7]. This may be due to aerotolerance characteristics, which contribute significantly to the aerobic transmission of *C. jejuni* to humans through food [8]. Reactions to stress, the condition of being viable but unable to be cultured, the stationary phase, and most critically, the production of biofilms [9–13] are all possible survival strategies for this pathogen. Bacteria may live for a long time in biofilms by entering a “dormant state” and maintaining metabolic activity while stopping cell division. A previous study indicated that *C. jejuni* biofilms may endure extreme conditions, which increase their infectiousness [14].

Because of their increased tolerance to chemical and physical stressors, biofilm-forming bacteria have significant consequences for pathogenesis [15]. That’s why getting rid of them is hard [16]. In addition, it is well-known that biofilm development at sensitive spots in poultry production facilities may contribute to the persistence of pathogens in chicken meat [17], and the dismantling of particular types of equipment utilized throughout different processing stages might be problematic. Due to the prevalence of these diseases, there has been a push for developing better sterilizing techniques for meat [18]. In Iraq, there is not as much research on *C. jejuni* infections as there is on other foodborne bacteria [19–23]. Therefore, there is an urgent need to conduct more studies on this pathogen, especially due to the large consumption of poultry meat, which is the primary source of campylobacteriosis. Due to the importance of ozone in the meat industry [24], it was used in this study to induce oxidative stress on *C. jejuni*.

This study was conducted to assess the influence of biofilm formation on *C. jejuni* survival rates under hostile conditions to understand the resistance of biofilm producers’ pathogens to various sterilizers, whether on the farm or in poultry production plants, which consequently cause contamination of meat with these pathogens.

## Materials and Methods

### Ethical approval

This study was approved by the Technical Institute of Suwaria at the Middle Technical University in Baghdad, Iraq (November 05, 2022). No human or animal subjects were included in this study. All procedures were performed in accordance with the accepted standards.

### Study period and location

This study was conducted from November 2022 to February 2023 in Al-Suwaira City, situated in the Wasit governorate in the middle east of Iraq.

### Bacterial strains

A total of 20 *C. jejuni* strains (eight from cecal contents and 12 from frozen chicken carcass rinse) obtained from a previous study by Ghaffoori [25] were subjected to the biofilm detection method. Isolation and identification of these strains were performed according to the standard bacteriological protocols described by previous studies [25–27]. employing tests for oxidase, catalase, H_2_S generation, microaerobic growth, and hippurate hydrolysis in addition to microscopic analysis of morphology and motility. Oxoid Biochemical Identification System Campy (Oxoid, ID0803M, United Kingdom) was used in further biochemical research to differentiate *Campylobacter*. The multiplex polymerase chain reaction assay was used to confirm the presence of *C. jejuni* strains, as previously reported by Ghaffoori [25].

### Culture conditions

Frozen single-use stocks that were preserved at −80°C in a double-strength nutrient broth (Oxoid, CM0001) containing 20% (v/v) of pure medical glycerin were thawed in a refrigerator overnight, subcultured onto modified Charcoal Cefoperazone Deoxycholate -Preston (mCCDA-PA, Oxoid, CM0739) agar, and incubated in an anaerobic jar under microaerophilic conditions using Campy Gen sachet (Oxoid, CN0035): 5% oxygen, 10% carbon dioxide, and 85% nitrogen at 37°C for 24 h.

### Biofilm assay using crystal violet (CV) staining

CV staining was used to measure biofilm formation for *C. jejuni*, as described previously by Reeser *et al*. [28] with some modifications. Cells from an agar plate were used to inoculate Mueller-Hinton broth (MHB, Oxoid, CM405B) and then grown overnight at 37°C under microaerophilic conditions as shaking cultures. Following overnight growth, cell culture in this broth was spectrophotometrically standardized on an optical density (OD)_600_ nm of 0.25. To allow biofilm formation, 24-well polystyrene Costar plates with flat bottoms (Corning, USA), including 1 mL of MHB, were injected overnight to an OD_600_ nm of 0.025, which corresponds to a final concentration of approximately 2.5 × 10^7^ colony-forming unit (CFU). Each isolate is tested in triplicate. Wells with only sterile MHB broth served as controls. The plates were incubated at 37°C in a microaerophilic atmosphere. After incubation, the medium in each well was carefully tapped out. The wells were washed 4 times with 0.2 cc of phosphate-buffered saline. This eliminates microorganisms floating in the air. Bacterial biofilms formed in the wells were fixed with 2% sodium acetate, allowed to dry for 30 min at 55°C, and then colored with CV for 5 min at 25°C, as described by Reeser *et al*. [28]. Two washes of deionized water removed the unbound resume. Bind CVs were decolored using 80% of 20% etha­nol and 20% acetone, and the wells were dried at 55°C for 15 min. The absorbance at 570 nm was measured spectrophotometrically, and 100 μL of this solution was taken from each well and plated on a 96-well plate. The study was conducted 3 times with identical results each time. Biofilm production was interpreted using Stepanović *et al*.’s [29] criteria, which classify strains according to their average OD in the context of this kind of computation, the OD c value should not be subtracted from the average OD value of the strain:

- OD ≤ OD c = no biofilm producer (0)
- OD c < OD ≤ 2 × ODc = Weak biofilm producer (+)
- 2 × ODc < OD ≤ 4 × ODc = Moderate biofilm producer (++)
- 4 × ODc < OD = strong biofilm producer (+++)

The final OD value of a strain is calculated by subtracting its average OD from its OD c (OD = Average OD of a strain - OD c).

OD c = Average OD of negative control + (3× Standard deviation [SD] of negative control).

### Survival rate of *Campylobacter* in a hostile oxidative-damaged environment

The oxidative stress tolerance of five different strains of *C. jejuni* was evaluated by plating 0.5 McFarland standard corresponding to 1.5 × 10^8^ CFU of each strain on m CCDA-PA agar plates and then exposing to gaseous ozone at two different exposure times (1, 2 min) as previously designated by Mohammed *et al*. [30] with some modifications. All plates were temporarily sealed into polythene bags and treated for 1 and 2 min with gaseous ozone using an ozone originator (A2Z/AQUA-6, USA) to release 600 mg/h of ozone gas. The treated dishes were removed from the bags after 25 min and incubated in an anaerobic jar under microaerophilic conditions at 42°C for 24 h, and viable colonies were counted.

### Determination of the number of viable cells

The number of live cells was determined on mCCDA-PA agar plates using the miles and mizra counting method. In brief, five drops (5 × 20 μL) of successive dilutions of a sample of the planktonic cells were placed on mCCDA-PA agar plates, dried, and incubated at 42°C for 2 days. CFU/mL was determined by counting the number of colonies using the following calculation:

CFU/mL = Average number of colonies for a dilution × 50 × dilution factor [31].

### Statistical analysis

MedCalc Software bvba version 18 (BE, USA) (https://www.medcalc.org/) was used to compute statistically significant differences. Proportion, mean, and standard deviation were employed as descriptive statistics. We used a two-sample Chi-square test to compare percentages and a t-test with 5% significance levels to compare means (Mean ± SD) for the chosen biofilm-producing bacteria.

## Results

**Figure-1:**
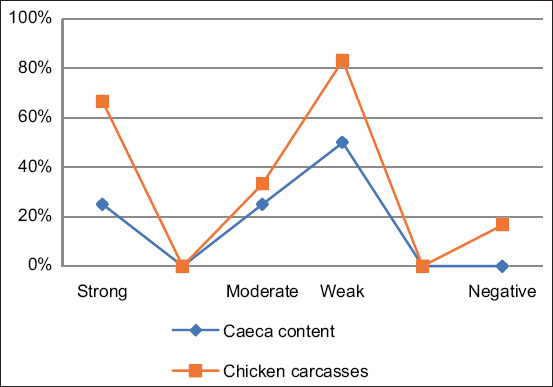
Biofilm production in *Campylobacter* strains based on their origin

Table-1 shows that the majority of strong biofilm-producing *Campylobacter* isolates were isolated from imported frozen chicken meat (25%), whereas a minority (10%) were isolated from caeca content. In addition, 10% of the tested isolates from the imported carcass rinse were not positive for this character. Moreover, strong biofilm production was detected in seven isolates (35%) (Table-2 and Figure-1), of which 25% and 41.7% were from caeca content and frozen chicken meat, respectively. Moderate and weak development of biofilms were detected in 3 (15%) and 8 (40%) isolates, respectively, whereas only 2 (10%) isolates were detected as negative for this characteristic. There was no statistically significant influence (p > 0.05) on the biofilm formation characteristics of these isolates (Table-2).

**Table-1:** Strains used in this study and their ability to produce biofilm based on sample origin.

Origin	Sample code	Source	Biofilm production
Live chickens	L1	Caeca content	+++
	L2	Caeca content	++
	L3	Caeca content	++
	L4	Caeca content	+
	L5	Caeca content	+++
	L6	Caeca content	+
	L7	Caeca content	+
	L8	Caeca content	+
Local broilers meat	LF1	Carcass rinse	++
	LF2	Carcass rinse	+
Imported broilers meat	IF1	Carcass rinse	+
	IF2	Carcass rinse	_
	IF3	Carcass rinse	+
	IF4	Carcass rinse	+++
	IF5	Carcass rinse	+++
	IF6	Carcass rinse	+++
	IF7	Carcass rinse	_
	IF8	Carcass rinse	+
	IF9	Carcass rinse	+++
	IF10	Carcass rinse	+++

+++=Strong, ++=Moderate, +=Weak, _=Negative; L=Isolates from caeca content; LF=Isolates from local frozen chicken meat; IF=Isolates from imported chicken meat

**Table-2:** Biofilm formation in *Campylobacter jejuni* isolated from caeca content and frozen carcasses.

Origin	Positive				Negative
	
	No. of the tested strains	n/N (%) strong	n/N (%) moderate	n/N (%) weak	n/N (%)
Caeca content	8	2/8 (25)	2/8 (25)	4/8 (50)	0/8 (0)
Chicken carcasses	12	5/12 (41.7)	1/12 (8.3)	4/12 (33.3)	2/12 (16.7)
Total	20	7/20 (35)	3/20 (15)	8/20 (40)	2/20 (10)
p-value		p = 0.4547 χ^2^ = 0.559	p = 0.3177 χ^2^ = 0.999	p = 0.4666 χ^2^ = 0.530	p = 0.2350 χ^2^ = 1.411

Our results (Table-3) revealed that after exposure to gaseous ozone at 600 mg/h for 2 min, strong biofilm-producing strains (L1 and IF6) exhibited a higher survival rate as they showed a limited reduction of 3 and 2 logs, respectively, whereas moderate and weak biofilm-producing strains (LF1 and IF8) exhibited a higher reduction of 4 and 5 logs, respectively. On the other hand, the negative biofilm production strain (IF2) displayed a limited survival rate with a reduction of 6 logs. A statistically significant difference (p < 0.05) in the rate of survival was observed between strong biofilm producer strains (t = 3.756, p = 0.0330) and moderate, weak, and negative biofilm producer strains after the same exposure times.

**Table-3:** Correlation of biofilm production of *Campylobacter jejuni* to their survival under oxidative stress after gaseous ozone (600 mg/h) exposure at two different exposure times (1, 2 min).

Sample code	Biofilm production	Log_10_ CFU/mL count before treatment	log_10_ CFU/mL count after treatment					Mean ± SD

			1 min	log_10_ decreased	2 min	log_10_ decreased	Total log_10_ decreased	
L1	Strong	1.5 × 10^8^	1.2 × 10^6^	2	1.1 × 10^5^	1	1	2.5 ± 0.5
IF6	Strong	1.5 × 10^8^	1.4 × 10^7^	1	1.4 × 10^6^	1	1	
LF1	Moderate	1.5 × 10^8^	1.3 × 10^5^	3	1.3 × 10^4^	1	1	5 ± 0.82
IF8	Weak	1.5 × 10^8^	1.1 × 10^5^	3	1.1 × 10^3^	2	2	
IF2	Negative	1.5 × 10^8^	1.4 × 10^4^	4	1.4 × 10^2^	2	2
								t = 3.756 p = 0.0330

CFU=Colony-forming units, L=Isolate from caeca content, IF=Isolates from imported chicken meat, LF=Isolate from local frozen chicken meat, Mean ± SD=Mean ± Standard deviation

## Discussion

Microbial biofilms are a serious problem in food processing industry. More and more evidence suggests that biofilms may assist *C*. *jejuni* in surviving in the food chain [32]. In fact, this microaerophilic pathogen is exposed to oxidative stress and other potentially harmful environmental factors as it moves through the food chain. Biofilm is a bacterial lifestyle that has been shown to confer resistance to a variety of threats, including antibiotics and the human immune system [32]. In this study, the capacity of these strains to form biofilms was investigated. The impact of the biofilm on the ability of isolates to withstand oxidative stress was also measured. Our results (Table-1) showed that the majority of strong biofilm producers (25%) were isolated from imported frozen chicken meat, whereas a minority (10%) were isolated from caeca content. Moreover, our results (Table-2 and Figure-1) showed that 50% were identified as strong to moderate biofilm producers.

*C. jejuni* may persist and live outside the host under circumstances that are deleterious to them by creating biofilms on plastics, stainless steel, and other materials that come into contact with food, which are ubiquitous in food processing settings or poultry house water systems. Human *C. jejuni* infections are common [2], and these biofilms may be a major source of the bacteria responsible for these infections. *C. jejuni* biofilms may form on a wide range of materials, including stainless steel, nitrocellulose, and glass fiber filters. Associating with food surfaces or organic components promotes the formation of biofilms [33]. It has been demonstrated that this pathogen may develop biofilms on many different surfaces; however, contradictory findings have rarely been reported. *C. jejuni* may adhere to and develop biofilms on hydrophobic surfaces, similar to polymers used in watering systems [28], whereas it cannot attach to polystyrene [34, 35]. These discrepancies might result from inherent changes across strains, or they could be the result of subtle methodological inconsistencies among experiments. Interestingly, when cultivated with *Escherichia coli* or *Pseudomonas aeruginosa*, *Campylobacter* was found in samples of retail food products that formed more biofilm than when grown alone [36]. *C. jejuni* multilocus sequence type ST-47, which is common in New Zealand and linked to poultry and humans, produces more biofilm when grown in an ecosystem with *Enterococcus faecalis* and *Staphylococcus simulans*. In addition, *C. jejuni* may find a hospitable environment in poultry processing plants due to the presence of several chicken-derived bacterial communities in the poultry ecosystem. According to our data, 25% and 41.7% of the isolates found in caeca material and frozen chicken flesh produced strong biofilms, respectively. It has been shown that adding *Brucella* broth to chicken flesh exudate encourages biofilm formation by *C. jejuni* on polystyrene, glass, and stainless steel [37]. The conditioning layer of chicken juice on abiotic surfaces facilitates biofilm development by providing an adhesive base on which *C. jejuni* biofilms can start and proliferate. This suggests that *C. jejuni* infestation may be exacerbated by substances released from meat exudates [37], among other foodborne pathogens, in an industrial food context. In unfavorable environments, such as the food chain or pipelines [37], conditioning, which is the growth of absorbed layers on a surface, may be visible as biofouling [38]. Biofouling is a significant issue in the food chain because it increases biofilm growth, decreases heat transfer efficiency, and impedes liquid movement inside pipes [37]. Therefore, rather than biofilm production, adhesion and survival on surfaces and in existing biofilms of other species are the most plausible methods for *C. jejuni* to remain in the food chain.

To assess their resistance to oxidative stress and aerotolerance, *C. jejuni* isolates were subjected to 600 mg/h of gaseous ozone for either 1 or 2 min. Table-3 shows that strong biofilm-producing strains had a greater survival rate after 2 min exposure than moderate and weak biofilm-producing strains, with a decrease of up to 5 logs. The survival rate of strong biofilm-producing strains significantly differed from that of moderate, weak, and negative biofilm-producing strains (t = 3.756, p = 0.0330) for the same period.

The ability of *C. jejuni* to withstand oxidative stress has been the subject of numerous scientific investigations. Chynoweth *et al*. [39] have reported that various *C. jejuni* strains isolated from various environments showed evidence of adaptation to atmospheric oxygen. When grown in microaerobic conditions (MAC) or aerobic acclimated conditions, the *C. jejuni* Bf strain had a better chance of survival than the *C. jejuni* NCTC 11168 reference strain when it was exposed to hydrogen peroxide (1 mM) [40]. Garénaux *et al*. [41] found that *C. jejuni* NCTC11168 populations dropped by 0.5 log10 after exposure to 500 μM paraquat for 1 h.

Other strains of *C. jejuni* have shown tolerance to oxidative stress generated by reactive oxygen species (ROS)-promoter chemicals [42, 43]. *C. jejuni*’s ability to survive in oxygen-rich environments is dependent on its resistance to oxidative stress brought on by the inevitable formation of harmful ROS during aerobiosis [44]. Microorganisms exposed to oxygen change genetically and physiologically as a defense mechanism against oxidative stress. *C. jejuni* cells subjected to the effects of oxidative stress undergo a morphological transition from a spiral to a coccoid shape [45]. The cells of this bacterium may also be filaments, doughnuts, and rods [46]. Similar findings have been observed in other investigations, where cells changed from spiral to coccoid shapes after exposure to air [46,47]. Reduced cell size and subsequent appearance of coccoid forms may be linked to changes in the expression pattern of mreB [40], which is essential to maintain the round shape of bacillary cells through the creation of intracellular helical filament. These changes may help these strains adapt and survive in adverse environments. PerR, CosR, and Fur are transcription factors that regulate the expression of genes involved in *C. jejuni*’s response to oxidative stress [44]. These genes include alkyl hydroperoxide reductase (ahpC), catalase (katA), and superoxide dismutase. *C. jejuni* biofilm growth is favored in the presence of oxygen, as previously reported by Reuter *et al*. [48]. Nonetheless, prior studies by Oh and Jeon [49] have shown the importance of oxidative stress in the development of *C. jejuni* biofilm in microaerobic environments. Mutations in genes involved in the defense against oxidative stress, most notably ahpC, greatly increase biofilm production under MAC, indicating a link between oxidative stress and biofilm growth in *C. jejuni*. Researchers have studied how biofilms affect the ability of cells to withstand oxidative stress. As oxidative stress increased rapidly during bacterial biofilm development, Wang *et al*. [50] discovered that virtually all metal transporter-related genes had been increased by 2–11-fold on Cu when comparing Cu to other substrates (1–2 fold for SS and Ti, 2–9 fold for Ni). In a similar vein, Oh *et al*. [51] concluded that *C. jejuni* biofilm growth is encouraged by oxidative stress in aerobic environments.

The enhanced generation of ROS in the ahpC mutant significantly aided biofilm growth in *C. jejuni* because the biofilm level recovered to that of the wild type after antioxidant treatment. Oh and Jeon [49], on the basis of Imlay [52], postulated that oxidative stress would have an effect on biofilm development even in aerobic environments. Aerobiosis always results in the production of ROS [52]. Living in low-oxygen conditions allows microaerophilic bacteria to reduce oxidative stress because the oxygen content within the cell is the same as that outside the cell [52]. The microaerophile *C. jejuni* is more vulnerable to oxidative stress than microaerophilic *C. jejuni* because it requires a low-oxygen concentration to grow. In previous research, ROS levels in *C. jejuni* were shown to be much higher under aerobic conditions than under MAC [51]. These results strongly suggest that ROS buildup is necessary for *C. jejuni* biofilm formation under aerobic conditions. Therefore, scientists expected that under aerobic conditions biofilms would modify the expression levels of genes involved in oxidative stress defense. Ozone was chosen to induce oxidative stress in *C. jejuni*. Although ozone is a strong oxidizing agent, its antibacterial activity decreases with biofilm-producing isolates. In addition, the effectiveness of ozone against bacteria is inversely proportional to the type of biofilm produced. These findings have a significant impact on public health since ozone is one of the alternatives used for the sterilization of meat, in particular poultry carcasses, in slaughterhouses (in chillers) as well as in food processing plants and restaurants. In addition, ozone-resistant bacteria may serve as important reservoirs of antimicrobial resistance in the environment. Therefore, these results can serve as a basis for understanding the resistance of bacteria to sterilizers (in particular ozone) used in slaughterhouses and food processing plants. Many features of *C. jejuni* biofilm production, including the ability to construct biofilms in the presence of ambient oxidative stress, require further investigation.

## Conclusion

Our results indicate that *C. jejuni*, a potent biofilm producer, is highly resistant to the oxidative damage caused by exposure to ozone. Therefore, *C. jejuni* varieties fare better under oxidative stress. *C. jejuni* resistance to oxidative stress may be a major factor in the pathogen’s ability to spread and persist in the environment, particularly during transmission to food preparation surfaces. The greater the ability of the pathogen to persist in its natural environment, the higher the risk that humans may be exposed to it. Our results showed that oxidative stress stimulates *C. jejuni* biofilm growth under adverse circumstances; however, the molecular intricacies of this process need to be elucidated in future research. Further research at the cellular level is required to obtain a comprehensive understanding of how *C. jejuni* is able to resist oxidative damage

## Author’s Contributions

MHGK: Study design, sample collection, laboratory work, analysis, and drafted and revised the manuscript. The author has read, reviewed, and approved the final manuscript.
